# Women Epidemiology Lung Cancer (WELCA) study: reproductive, hormonal, occupational risk factors and biobank

**DOI:** 10.1186/s12889-017-4191-1

**Published:** 2017-04-17

**Authors:** Isabelle Stücker, Diane Martin, Monica Neri, Pierre Laurent-Puig, Hélène Blons, Martine Antoine, Anne Guiochon-Mantel, Sylvie Brailly-Tabard, Marianne Canonico, Marie Wislez, Jean Trédaniel, WELCA study group, I. Stücker, J. Trédaniel, M. Wislez, M. Antoine, A. Arame, J. Arrondeau, A. Badia, M. H. Baud, M. Benhadria, C. Bernard, H. Blons, C. Boffa, H. Boudabous, G. Bousquet, S. Brailly-Tabard, S. Brosseau, O. Bylicki, J. Cadranel, J. Camuset, M. Canonico, N. Carlier, S. Cénée, J. Chapron, T. Chinet, A. Chohra, K. Chouahnia, C. Chouaid, P. Combe, P. Crequit, H. De Jacquelot, C. Delmas, H. Doubre, L. Doucet, B. Duchemann, C. Dumenil, D. Dusser, C. Epaud, E. Fabre, V. Fallet, M. Febvre, S. Fraboulet, T. François, N. Frémont, S. Friard, C. Gazaniol, F. Giraud, P. Giraud, V. Giraud, E. Giroux-Leprieur, V. Gounant, A. Guiochon-Mantel, L. Hajouji Idrissi, I. Honoré, T. Issoufaly, L. Jabot, J. L. Jagot, A. Jouan, A. Jouinot, S. Jouveshomme, S. Labrune, M. Lafay, P. Laurent-Puig, A. Lavole, H. Le Floch, C. Le Maignan, F. Le Pimpec-Barthes, A. Legras, A. Lenfant, M. Licois, A. Lurie, J. Margery, D. Martin, M. A. Massiani, M. Matrat, A. C. Métivier, G. Meyer, C. M. Monnet, I. Monnet, C. Mulot, J. M. Naccache, C. Naltet, M. Neri, M. C. Pailler, J. Pastre, N. Pécuchet, C. Pedrero, G. Plu-Bureau, D. Pouessel, C. Pricopi, M. Prosper, E. Rivaud, F. Rivière, L. Rosencher, G. Rousseau-Bussac, N. Rozensztajn, A. M. Ruppert, M. Sahut d’Izarn, Y. Salles, S. Salmeron, M. Sanchez, C. Thibault, F. Vaylet, T. Vieira, F. Vinas, G. Zalcmann

**Affiliations:** 1 Université Paris-Saclay, Univ. Paris-Sud, UVSQ, CESP, Inserm UMRS1018, 94807 Villejuif, France; 2 Université Paris Descartes, Inserm UMR 5775 EPIGENETEC, 75006 Paris, France; 3 Inserm UMR-S1147, université Paris Sorbonne Cité, 75006 Paris, France; 4 Hôpital Européen Georges-Pompidou (HEGP), Assistance publique-Hôpitaux de Paris, 75015 Paris, France; 5 Service d’Anatomie pathologique, AP-HP, Hôpital Tenon, Paris, France; 6 Service de Génétique moléculaire, Pharmacogénétique et Hormonologie, Hôpital de Bicêtre, APHP, Le Kremlin-Bicêtre, France; 7 Inserm UMR S1185, Faculté de médecine Paris sud, Université Paris sud, Université Paris Saclay, Paris, France; 8 Service de Pneumologie, AP-HP, Hôpital Tenon, Sorbonne Universités, UPMC Univ. Paris 06, GRC-04, Théranoscan, Paris, France; 9 Université Paris Descartes, Unité de cancérologie thoracique, Groupe Hospitalier Paris Saint-Joseph, Paris, France

**Keywords:** Biological specimen banks, Case-control studies, Estrogens, Female, Lung neoplasms, Molecular epidemiology, Occupational exposure, Reproductive history, Surveys and questionnaires

## Abstract

**Background:**

Lung cancer aetiology and clinical aspects have been mainly studied in men, although specific risk factors probably exist in women. Here we present the rationale, design and organization of the WELCA study (Women Epidemiology Lung CAncer) that has been launched to investigate lung cancer in women, focusing particularly on hormonal and occupational factors.

**Methods/Design:**

WELCA is a population based case-control study and planned to recruit 1000 cases and 1000 controls in three years, based on study power calculation. Eligible cases are female patients newly diagnosed with lung cancer, living in Paris and the Ile de France area and aged up to 75 years. Almost all Parisian pneumology and oncology clinical departments are involved. The control group is a random sample of the population living in the same area, frequency-matched on age and additionally stratified on the distribution of socio-professional categories of women residing there. After acquisition of written consent, research nurses administer standardized computer assisted questionnaires to all the subjects in face-to-face interviews and acquire anthropometric measures. Besides usual socio-demographic characteristics, information is gathered about menstrual and reproductive factors, hormonal treatments, lifestyle and leisure characteristics, occupational history, personal and familial medical history. Biological samples are also collected, in order to establish a biobank for molecular epidemiology studies. Molecular characteristics of the tumours will be obtained and patients will be followed up for five years.

**Discussion:**

The WELCA study aims to answer key questions in lung cancer aetiology and clinical characteristics specifically in women. The role of hormonal impregnation is investigated, and the interactions with cigarette smoking or body mass index (BMI) will be analyzed in detail. The occupational history of the subjects is carefully reconstructed, focusing in particular on the service sector. The creation of a biobank for collection of serum, plasma, DNA and tumour tissue will allow the genetic and biochemical characterization of both the subjets and the tumours. The follow-up of the patients will help in disentangling the role of hormonal factors and tumour molecular characteristics in survival.

## Background

Knowledge about lung cancer aetiology and clinical aspects has been gained from studies that included mainly men, because of its rarity among women until some decades ago. The pathology of lung cancer in men and women is the same, but important differences between genders exist and remain largely unexplained, suggesting the existence of specific risk factors in women [1–5]. The clinical features of lung cancer in women are different from those in men in terms of histological type, survival, tobacco exposures and treatment responses [6, 7]. Squamous cell histologic type still remains the most common cancer in ever smoking men, while lung cancer in smoker women is predominantly adenocarcinoma [8, 9]. From a prognostic point of view, women have better survival rates than men (e.g in France, 20% survival at five years vs. 16% survival for men) [10]. Molecular signatures, like mutations in the *EGFR* gene and *ALK* translocation in adenocarcinomas, have been found to be differently distributed among female and male patients [11].

Smoking is by far the major risk factor for lung cancer. However, a large percentage of adenocarcinoma in women, between 20% and 30% in Western countries and up to 80% in Asian countries, occur without a history of smoking, highlighting the existence of unrecognised risk factors, possibly women specific. Controversy still exists about the possible increased susceptibility to the carcinogenic effects of tobacco smoke in women compared to men [12]. This debate led to research into the role of reproductive and hormonal factors in female lung cancer.

Occupational exposures are the second major risk factor for lung cancer, and the attributable risk fraction is approximately 15% to 30% in men and 3 to 6% in women [13]. However, the occupational lung carcinogens that have been identified thus far are found almost exclusively in male occupations (e.g., building, metallurgy, mining). Occupational epidemiology research still suffers from androcentrism, as demonstrated by the fact that around half of recent studies recruited only male participants, and the gender factor was not thoroughly evaluated in most mixed studies [14, 15]. Very few studies have investigated the existence of specific occupational risk factors in female occupations [16, 17].

Overall, findings in the literature often show wide discrepancies, which may be due at least in part to the heterogeneity of lung tumours, insufficiently taken into account by histological type. Molecular characterization of tumours may help in better disentangling the role of risk factors in lung cancer development, progression and survival.

### General and Specific Objectives

WELCA (Women Epidemiology Lung CAncer) is a population based case-control study aimed to investigate the aetiology of lung cancer in women, focusing especially on hormonal and occupational factors.

The first objective of the study regards the role of female hormones, including lifetime endogenous exposure and exogenous hormonal treatment. The history of women hormonal impregnation is reconstructed by collecting information from the subjects about traditional reproductive factors, hormonal treatments, and diseases or clinical indicators related to a hormonal modification. The creation of a biobank for collection of serum, plasma, DNA and tumour tissue allows the genetic and biochemical characterization of both the subjets and the tumours. The interactions between hormonal and reproductive factors and cigarette smoking or body mass index (BMI) will also be analyzed in detail.

The second objective is the investigation of occupational risk factors. Occupational history of the subjects is carefully reconstructed in face-to-face interviews with structured questionnaires, focusing in particular on the service sector (e.g. household cleaning, laundry, hairdressing and catering).

Patients will be followed up for five years, in order to study the role in survival of hormonal factors and tumour molecular characteristics.

### Female hormones

One way to explore gender differences in lung carcinogenesis is to analyse the role of female hormones, and particularly oestrogens.

Experimental, clinical and epidemiological studies indicate that oestrogens may play a role either by a direct promoting effect of lung carcinogens on cell proliferation or by an indirect influence, e.g. polycyclic aromatic hydrocarbons (PAHs) present in tobacco smoke may stimulate oestrogen metabolism [5, 18–21]. Receptors of sex steroids, including oestrogens, progesterone and androgens, have been found in bronchial and alveolar epithelia and in airway smooth muscle, and enzymes producing sex steroids have been observed in lung parenchyma [22, 23].

However, studies investigating the relationship between indicators of lifetime endogenous exposure and the incidence of lung cancer in women (e.g. age at puberty, age at menopause, parity, menopausal status) have brought suggestive but no clear information to date. The effects of female hormonal and reproductive factors are difficult to detect, possibly because they interact with other host genetic or environmental factors in complex ways that still await to be disentangled [24, 25].

**Smoking** is related with an anti-estrogenic activity, as suggested by an early age at menopause and an increased risk of osteoporosis among female smokers, and therefore it is a major confounding factor [26]. Analyses of reproductive factors are not frequently stratified on smoking status, although smoking prevalence may differ greatly in different populations, e.g. in Asians compared to North Americans. On the other hand, adjustment for smoking usually does not take into account properly the three main components of cigarettes smoking as regards lung cancer risk: duration, amount, and time since quitting [27].

**BMI** is another important parameter to consider, as fat tissue is involved in the production of oestrogens in women, particularly after menopause. BMI is also associated with menstruations, and low BMI, as observed in eating disorders, is often accompanied by amenorrhea. The role of BMI has been rarely considered in previous studies on reproductive factors and lung cancer, yet a higher BMI has been reported to be associated with a reduced risk of lung cancer in current and former smokers [28].

Most epidemiologic studies investigating the risk of lung cancer in women using **exogenous hormonal treatments** showed conflicting results. A protective effect of menopausal hormone therapy (MHT) and oral contraceptive (OC) use has been proposed in a pooled analysis [29], and reduced lung cancer risks in MHT users respect to never users have been found in a meta-analysis of case–control studies [30]. However, a meta-analysis including studies with diverse design found instead a positive association of MHT use with lung cancer in specific subgroups of subjects [31], and most studies on OC administration found no association [29].

The type of hormonal treatment has been very rarely distinguished. In the case of MHT, for example, studies showed conflicting results when oestrogen alone or oestrogen + progesterone treatments were investigated [32–34]. The chemical composition of HTM varies by country: in France it has been used predominantly an estrogen molecule identical to the natural estradiol, administered transdermally, while in Anglo-Saxon countries oral oestrogen of equine origin has been prescribed in most cases. Similarly, natural progesterone is widely used in France, while in Anglo-Saxon countries a synthetic progestin (medroxyprogesterone acetate) is preferred. Previous works suggested that these differences may partly explain the divergent results between France and the United States regarding the risk of several estrogen-dependent pathologies, such as breast cancer [35, 36] or cardiovascular diseases.

Moreover, specific molecular and biological **tumour characteristics** could play a role. For example, the interaction of expression levels of hormonal receptors or *EGFR* mutations with hormonal treatment have never been investigated in relation to lung cancer, to our knowledge.

### Occupational exposures

As reported previously, very few studies have been devoted to the investigation of occupational risk factors of lung cancer among **females** compared to the number of studies conducted among men. While in the past only few women were engaged in a professional activity, currently the vast majority of women hold or have held an employment throughout their adult lives (68% according to the 2015 survey of the French National Institute of Statistics and Economy Studies (INSEE) [37]. Women’s occupations are different from men’s, involve particular exposures that could be associated with an increased risk of lung cancer and deserve specific studies in order to fill a real research gap.

Studies that have systematically investigated this issue found excesses of lung cancer associated with **general services** (nurses, maids, hairdressing, laundry, catering) [38–41]. A meta-analysis of cancer risk associated with the profession of hairdresser showed a 30% increase in the risk of lung cancer associated with this activity [16]. Numerous chemicals are present in hair salons, and the International Agency for Research on Cancer classified the associated profession into group 2A (probably carcinogenic to humans) without identifying a particular substance [42].

In China, the repeated exposure of women to **cooking fume exposure or indoor use of coal for heating homes** has been frequently associated with an increased risk of lung cancer and likely accounts for a percentage of the high incidence of lung cancer among Asian non-smoking women [43]. A Chinese study on restaurant cooks revealed a higher level of oxidative stress in association with exposure to cooking fumes among women than men [44]. These fumes are generated when cooking food at high temperatures in oil, whose degradation produces particles, PAHs, aromatic amines, and nitro-PAH. The carcinogenic effects of some of these molecules have been shown, indicating the need to investigate whether women working in restaurants or in fast food establishments have a high risk of lung cancer. Studies evaluating exposure levels showed that the concentration of ultrafine particles is high in restaurant kitchens [45]. The role of particles in lung carcinogenesis is practically certain and ultrafine particles are the object of great interest in the scientific community, after their contribution has been considered to be probable by a panel of experts [46].

The role of **solvents** in lung cancer is currently debated. In 1995, the International Agency for Research on Cancer (IARC) classified substances used in textile dry cleaning into group 2B (possibly carcinogenic to humans) [47]. Perchloroethylene, which was particularly used in dry cleaning, was classified into group 2A in 1995. A Swedish study conducted in a cohort of dyers and dry cleaners showed a significant 30% increase in the risk of lung cancer compared to expected frequencies, and the increase was stronger in women than men [48]. Similarly, two case-control studies found an increased risk in lung cancer related to perchloroethylene exposure [49, 50].

Occupational exposure to **asbestos** is a well recognized risk factor of lung cancer. Asbestos exposure has been always much more common in male-dominated occupations (e.g. insulators, ship builders, construction workers), however women also may have been exposed. For example, 64% of men and 23% of women were found to have a non-null probability of exposure to asbestos in a large case-control study conducted in France [50].

## Methods

### Study design

**Fig. 1:**
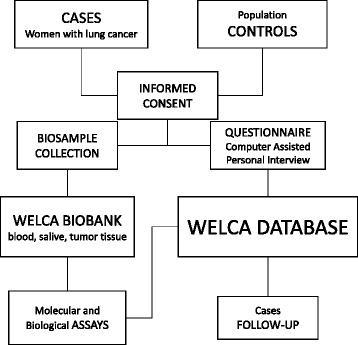
Schematic representation of the WELCA study organization

We have set-up a multi-centre population based case-control study. WELCA population includes women up to 75 years old living in Paris or in the Ile de France area (counties 75, 91, 92, 93, 94, 95, 77), regardless of their nationality. The study will recruit 1000 lung cancer incident cases and 1000 population-based controls during a three years period. The study organization is schematized in Fig. 1.

### Case selection

WELCA study involves almost all Parisian pneumology and oncology clinical departments. All women diagnosed with incident histologically confirmed primary cancer of the lung [ICD 10th revision: C34.0-C34.9] or trachea [ICD 10th revision: C33] are eligible to participate in the study. All histological types are considered, except carcinoid tumours. The case identification is performed at each ward by personnel participating in the multidisciplinary coordination meetings (MCM), during which all patients reports are routinely discussed.

Physicians inform the eligible patients and, if the written consent is obtained, arrange the blood sampling. They inform the patient that the interview will be carried out by a clinical research nurse who will contact her soon. The research nurse proposes the time and place of interview to the patient and sends her a written document to prepare the interview.

### Control selection

The control group is a random sample of the population residing in the study area. A polling institute with extensive experience contacts the women through random digit dialing, a procedure that gives each number (including unlisted numbers) an equal probability of being sampled. The list of numbers generated is then sorted to exclude business numbers or unassigned numbers. Each number is called 15 times before being abandoned as not answered. Calls are made in the morning or in the evening on weekdays and during the day on Saturdays, so that each woman has the same probability of being contacted. The selection of controls is conducted every two months and about 60 women are recruited each time.

Controls are frequency matched for age and county of residence with the cases. Furthermore, to minimize selection bias, controls are additionally stratified on the distribution of socio-professional categories of women residing in Paris/Ile de France, according to INSEE: workers, businesswomen/craftswomen/shopkeepers, managerial and professional occupations, intermediate professions, employees, farmers. Women who are without professional activity when contacted (retired, unemployment, disease leaving, maternity, cessation of professional activity) are classified according to their last job. A specific category is intended for women who never worked.

When an eligible subject is contacted by the polling institute investigators, the main objectives of the study and terms of participation are explained and agreement to participate is sought. Women who agree are informed that a research nurse will contact them in the next days. After making an appointment, the nurse sends the women the same preparatory written document as for the cases. At interview, the nurse collects the written consent to participate and then proceeds with the questionnaire administration and the biosample collection (blood or saliva).

### Organization

A steering committee including the principal investigator, the study monitor and two pneumo-oncologists (JT, MW) has been set up to facilitate the implementation and the proper conduction of the study. A clinical coordinator (JT) meets the research nurses every 2–3 months, in order to maintain their training and to follow the report of clinical characteristics of the cases (e.g. treatment, driver mutations, histology). The research nurses have been specifically hired for the study and have been trained particularly regarding lung cancer characteristics, eligibility criteria and the use of the computerized questionnaire. The study monitor meets them every two weeks to follow the case and control recruitment. A newsletter is sent by the study monitor to each partner monthly to follow the number of inclusions by centre and to maintain their implication in the study.

### Data and sample collection

#### Questionnaire

Interviews are conducted face to face at home of the subjects by the research nurses, using a CAPI procedure (Computer Assisted-Personal Interview), and last one to two hours. Interviewers daily transfer the completed questionnaires to the polling institute, which returns SAS files.

The standardised questionnaire (available on request) allows the collection of usual socio-demographic characteristics (including residences during the last 20 years, marital status and ethnic origins), reproductive and hormonal history, personal and family medical history, full professional history, lifestyle and recreational physical activity.

Data concerning *hormonal history* include age at puberty, regularity and duration of menstrual cycles during adult life, contraceptive methods used, age at menopause and adverse effects, history of oophorectomy. Data on exogenous hormones use are gathered by detailed questions on OC, possible treatments for infertility and HTM, including the brand of drugs used (with paper images list support), and the beginning and end date of each period.

*Reproductive factors* are collected via questions on pregnancies (number and outcomes), children (number and sex), age at first birth and infertility (if appropriate). In addition, women are requested to give the name of their doctor gynaecologist and/or GP, to be contacted in order to enable validation studies on the reported consumption and brands of OCs and/or MHTs.

Questions about *medical history* are focused on history of cancer and lung diseases, including infections (like pneumonia or tuberculosis), and fibroses linked to carcinogenic exposures (asbestosis, silicosis). The history of cancer in first-degree relatives of the subjects is also collected.

For each subject, height is collected and weight is measured. Changes in weight during adulthood by decade are also recorded, both in kilograms and size clothing.

Data on *smoking habits* are carefully gathered and include cigarette smoking, with beginning and end dates, together with quantity per day and type of cigarettes (blond or brown tobacco, filtered or not, brand) for each smoking episode. Specific questions are dedicated to smoking of cannabis, with quantity per day, beginning and end dates, and to passive smoking at work or at home.

The *occupational questionnaire* includes general questions on work history and, for each job, information on the activity and products of the company, a description of the tasks performed (nature, frequency, equipment used) and a description of the work environment. In addition, specific questionnaires have been developed concerning tasks, jobs and sectors that are frequently found or are considered of particular interest for the study: house cleaning, dry cleaning and laundry, hairdressing, catering and cooking, health, beauty therapist, nail salons.

Finally, for each job interviewers collect an accurate description of the working hours, the duty cycle over a weekly period, and the number of working days per week, in order to be able to consider troubles in circadian rhythm.

#### Clinical data and follow up

Research nurses collect clinical characteristics and vital status of the cases in medical records every 6 months for 3 years and then every year for 2 years. Information about diagnosis and medical care are gathered such as tumour localization, presence of metastasis, histological type, molecular profiling of tumours if available, stage and TNM classification, therapeutic decisions and responses.

#### Biobank

A centralized biobank for molecular epidemiology studies is established to collect and store blood or saliva from all the subjects and tumour tissue from the patients.

A 20 ml peripheral blood sample is taken by venipuncture from each subject (both cases, preferably before any treatment, and controls) and treated in local laboratories. All procedures follow a written standardized protocol that has been expressely developed. Briefly, blood is transported at 4 °C to the laboratory. The sample collected into two serum separator tubes with gel is allowed to clot for at least 60 min and, after spinning for 15 min at 4 °C and 3000 rpm in a standard centrifuge, serum is aliquoted in 0.5 ml cryotubes and stored at −80 °C. Similarly, blood collected into one tube containing EDTA additive is spun in the same conditions, then plasma and buffy coat (for DNA extraction) are carefully removed separately and aliquoted. Aliquots are periodically transported in dry ice to the biobank for final storage at −80 °C and subsequent analyses.

In case subject refuses blood sampling, a collection of saliva with an Oragene kit is proposed. Kits are stored at room temperature in the biobank until DNA extraction. The refusal to provide a blood or saliva sample will not constitute grounds for exclusion from the study. Specimens are labeled with date of sampling and a numeric code that is unique for each subject.

Serum and plasma are used for analysis of hormonal levels (e.g. FSH, 17ß-oestradiol, Sex Hormone-binding globulin, testosterone) and other circulating biomarkers.

DNA of the subjects will be extracted from leukocytes in the buffy coat or from saliva for the identification of genetic susceptibility factors in future projects.

The study monitor is in charge of the collection of biopsies or tumour specimens, contacts the pathology departments and organizes the transport of paraffin blocks and slides to the biobank. Tumour tissue is used for immunohistochemical studies on hormonal receptors. In the framework of a molecular study, all tumours will be analysed using targeted next generation sequencing (hotspotcancer panel V2, iontorrent technology, proton sequencing system). In addition, the availability of tumour tissue could allow to detect the presence of viruses that are suspected to play a role in lung cancer development, like HPV [51].

### Study power

Minimum ORs that may be detected with 1000 cases and 1000 controls, a power of 80% and a 5% level of significance (two-tailed test) are 2.67, 1.67, 1.47, 1.39, 1.31 for exposures whose prevalence in controls are 1%, 5%, 10%, 15% and 30% respectively.

### Regulatory aspects

In France, a global ethics approval based on the list of participating centres is sufficient for epidemiological studies that are not clinical trials, and it was obtained from the Institutional Review Board of the French National Institute of Health and Medical Research and by the French data Protection Authority (IRB-Inserm, no. 3888 and CNIL no. C13–52). The Protection to Persons Committee gave a favourable opinion about the biological collection, which has been authorized by the Ministry of higher education and research (CODECOH N° DC 2014–2078).

Each subject (case or control) has a code number which is used for the identification of the blood sample and the questionnaire instead of any nominative information. This procedure guarantees the confidentiality of personal data, the respect of regulatory requirements and the study blindness. The research team keeps the link between identification number and the subject’s name.

## Discussion

WELCA is a large population based case-control study designed to assess the role of hormonal and reproductive factors as well as occupational exposures in lung cancer among women.

Several aspects make the WELCA study innovative and competitive. We were able to include the clinical wards that treat the vast majority of lung cancer patients in Paris/Ile de France area. Having limited the study to a single large region densely populated like this is an asset to the study, first, because the number of eligible female lung cancer patients is high, and secondly because the collection of biological samples, including tumour material, is made easier.

The study is presented to the patients by their physician to maximize their participation and interviews of consenting women are conducted shortly after, in order to minimize survival bias in the participation in the study.

Control selection is always a matter of concern with this design. In order to minimize selection bias, we built up a control group taken from the general population, following an incidence density sampling, frequency matched on age of the cases and additionally stratified on socio-economic status of the women residing in the recruitment area.

Recall bias may not be ruled out completely in case-control studies, but it can be considered minimal in WELCA because data are collected by the same research nurses for cases and controls, using a standardized questionnaire and in the same conditions for both cases and controls.

Lung cancer prognosis remains discouraging among women. To reduce lung cancer incidence and mortality, it is important to identify the risk factors for lung cancer in order to implement proper prevention strategies. The high prevalence of women on hormone therapy (for birth control or menopause) requires the urgent development of studies to elucidate its relationship with lung cancer. Occupational exposures to carcinogens play a major role in the aetiology of lung cancer. Very few studies have examined the exposures in jobs that are typically performed by women to date. Professional activity is an area where prevention is possible. Thus, it is urgent to identify those situations that could cause an increased risk of lung cancer and to establish adequate prevention strategies.

The biological samples collected for the project (serum, whole blood, saliva, tumour tissue) together with the questionnaire will allow to set up innovative molecular epidemiology projects, specifically on lung carcinogenesis and susceptibility. One of the aims will be to relate tumour mutations to hormonal and reproductive factors as well as occupational and smoking history, an issue that has never been investigated, to our knowledge.

## Abbreviations

MHT: Menopausal hormone therapy
OC: Oral contraceptive
WELCA: Women Epidemiology Lung CAncer
